# Kif4 Is Essential for Mouse Oocyte Meiosis

**DOI:** 10.1371/journal.pone.0170650

**Published:** 2017-01-26

**Authors:** Nicole J. Camlin, Eileen A. McLaughlin, Janet E. Holt

**Affiliations:** 1 School of Environmental and Life Sciences, University of Newcastle, Callaghan, NSW, Australia; 2 Priority Research Centre for Reproductive Science, University of Newcastle, Callaghan, NSW, Australia; 3 School of Biological Sciences, University of Auckland, Auckland, New Zealand; 4 School of Biomedical Sciences and Pharmacy, University of Newcastle, Callaghan, NSW, Australia; Institute of Zoology Chinese Academy of Sciences, CHINA

## Abstract

Progression through the meiotic cell cycle must be strictly regulated in oocytes to generate viable embryos and offspring. During mitosis, the kinesin motor protein Kif4 is indispensable for chromosome condensation and separation, midzone formation and cytokinesis. Additionally, the bioactivity of Kif4 is dependent on phosphorylation via Aurora Kinase B and Cdk1, which regulate Kif4 function throughout mitosis. Here, we examine the role of Kif4 in mammalian oocyte meiosis. Kif4 localized in the cytoplasm throughout meiosis I and II, but was also observed to have a dynamic subcellular distribution, associating with both microtubules and kinetochores at different stages of development. Co-localization and proximity ligation assays revealed that the kinetochore proteins, CENP-C and Ndc80, are potential Kif4 interacting proteins. Functional analysis of Kif4 in oocytes via antisense knock-down demonstrated that this protein was not essential for meiosis I completion. However, Kif4 depleted oocytes displayed enlarged polar bodies and abnormal metaphase II spindles, indicating an essential role for this protein for correct asymmetric cell division in meiosis I. Further investigation of the phosphoregulation of meiotic Kif4 revealed that Aurora Kinase and Cdk activity is critical for Kif4 kinetochore localization and interaction with Ndc80 and CENP-C. Finally, Kif4 protein but not gene expression was found to be upregulated with age, suggesting a role for this protein in the decline of oocyte quality with age.

## Introduction

The fidelity of chromosome segregation during meiosis is key to producing high quality oocytes, capable of creating healthy offspring. In mammalian oocytes, errors in chromosome separation result in embryonic aneuploidy, which may lead to spontaneous abortion or trisomy births such as Downs Syndrome. A known risk factor associated with oocyte aneuploidy is maternal ageing, with the aneuploidy incidence approximately 2% for women in their 20s increasing to 35% for women in their 40s [1–3]. In recent years, a growing body of evidence suggests that alterations in chromosome architecture with age results in abnormal kinetochore structure, aberrant kinetochore microtubule (KT-MT) interaction and ultimately aneuploidy [4–6]. To further elucidate how such events arise, we investigated a kinesin motor protein, Kif4 which has known roles in microtubule flux [7].

The kinesin motor protein Kif4 (human homologue KIF4A) is a member of the Kinesin-4 subfamily, consisting of KIF4A and B in humans and Kif4 in mice [8]. Studies in mitotic systems have revealed key roles for Kif4 in chromosome condensation and separation, metaphase and midzone spindle formation as well as cytokinesis [7,9–18]. Such functions are achieved through the ability of Kif4 to interact with condensin I, influence kinetochore protein loading, regulate microtubule length throughout metaphase and telophase and control microtubule kinetochore flux [7,9,11,19]. A growing body of evidence suggests that Kif4 function is mediated by cell cycle kinases Cyclin-dependent kinase 1(Cdk1) and Aurora Kinase B (AurB) [14,19–21]. Loss of Kif4 has also been associated with multiple tumours and is evident in cancer cell lines, indicating an important role for this protein throughout the cell cycle [18,22,23].

To date the role of Kif4 in mammalian fertility has not been investigated. However, the *Drosophila* homologue Klp3A is essential for male and female fertility [24,25]. In males, mutations in Klp3A results in midzone instability and cytokinesis failure in meiotically dividing spermatocytes [24]. In contrast, females are able to successfully complete meiosis, however, oocytes are unable to support embryogenesis [25,26]. In meiotic *Xenopus* egg extracts, the *Xenopus* homologue XKlp1, limits microtubule growth and is important for correct spindle shape [27,28]. Thus, in the present study we sought to characterize the localization and role of mammalian Kif4 in female meiosis. Additionally, we present evidence indicating Kif4 is involved in kinetochore dynamics under the control of AurB/C and Cdk1.

## Materials and Methods

All reagents were obtained from Sigma-Aldrich unless otherwise specified.

### Animals and ethics approval

Animal use for this study was approved by the University of Newcastle Animal Care and Ethics Committee. C57BL/6 x CBA F1 hybrid cross were obtained from the University of Newcastle Animal Resources and housed with ad libitum water and food under a 12hrs light: 12hrs dark cycle.

### Oocyte collection and maturation

4–6 week old female mice were intraperitoneally (i.p) injected with 7.5 IU of pregnant mares’ gonadotropin (Intervet) 44-52hrs prior to a second i.p injection of 5 IU human chorionic gonadotropin (Intervet) or germinal vesicle (GV) oocyte collection from the ovary. 12hrs after the second injection MII stage eggs were collected from the ampulla. Oocytes were collected into M2 media containing BSA. For GV collections media was supplemented with 1mM milrinone (M4659) to prevent meiotic resumption. Cumulus cells were then removed enzymatically with hyaluronidase (300μg/ml; H4272) for MII or mechanically via repeated pipetting for GV oocytes. For collection from aged (12–19 months) females and corresponding young controls, GV oocytes were retrieved directly from the ovary without prior hormonal stimulation.

To allow meiotic maturation in vitro, GV oocytes were washed into milrinone free M2 media at 37°C (GVB/MI) or MEM (MII; 11900024, Gibco) with 20% FCS at 37°C in 5% CO_2_. GV oocytes underwent IVM for 1.5hrs for GVB stage, 7.5hrs for MI stage or 16hrs for MII stage oocytes. For inhibitor experiments 10μM ZM447439 (2458, Tocris), 100μM Roscovitine (R7772), 100nM BI 2539 (S1109, Selleck) or DMSO (0.2% final concentration; D2650) was added to media at 3.5hrs post washout. Inhibitor experiments were performed in triplicate with oocytes from age matched individual animals.

### Immunocytochemistry and proximity ligation assay

For all immunocytochemistry oocytes were fixed in 2% paraformaldehyde in PBS with 0.5% triton X for 30mins. Oocytes were blocked in 7% goat serum PBS-0.1% Tween for 1hr prior to overnight incubation with primary antibody (see Supplementary S1 Table). Secondary antibodies were conjugated with Alexa-488, Alexa-555 and Alexa-633 (see Supplementary S1 Table) and incubated with oocytes for 1hr at room temperature prior to Hoechst (20μg/ml) counter staining and mounting in Citifluor (Citifluor Ltd). All immunocytochemistry was performed in triplicate with oocytes from age matched individual animals. Secondary only negative controls were also performed to confirm the specificity of immunocytochemistry.

Proximity ligation assay (DUO92101; PLA) was performed as per manufactures instructions to detect proteins within 40nm of each other, and therefore potential protein-protein interactions. Oocytes were then counterstained in Hoechst and mounted in Citifluor as above. Specificity of PLA was confirmed through the use of negative controls including Kif4 antibody only, secondary antibody only or Kif4 antibody with testis specific protein Piwil1 antibody. All PLA was performed in triplicate with oocytes from aged matched individual animals.

Imaging was performed using an Olympus FV1000 confocal using a 60x/1.2 NA UPLSAPO oil immersion objective lens (Olympus, Australia). ImageJ (freeware; National Institute of Health) was used to measure fluorescent intensity and count PLA foci as previously described [29]. Briefly, the area and integrated density of each oocyte was measured. The average of the mean background was determined for secondary antibody only probed oocytes. Fluorescent intensity for each oocyte was then determined by subtracting the oocytes area times the mean background from the integrated density prior to normalizing to 1. For PLA ImageJ particle counting function was used to determine number of PLA foci per oocyte.

### Oocyte qRT-PCR

Five denuded oocytes were used per reaction. The zona pellucida was removed with Acid Tyrodes solution (T1788) prior to being washed in PBS/ polyvinylpyrrolidone. Following lysis, cDNA was prepared using the TaqMan Gene Expression Cells-toC_T_ Kit (AM1728, Ambion) as per manufactures instructions. A whole cDNA sample was used per one reaction. qRT-PCR was performed with a Light Cycle 96 SW 1.1 (Roche) using TaqMan mRNA assay (4369016, ThermoFisher Scientific) as per manufactures instructions. Ppia was employed as an endogenous control to normalize the expression levels of Kif4. The relative expression levels of Kif4 were calculated using the ΔCt method.

### Oocyte microinjection

GV oocytes were microinjected as previously described by Holt et al., with either Kif4 knock-down or mis-match morpholino oligo (Gene-tools; see Supplementary S2 Table for targeting sequence) [30,31]. Oocytes were then incubated in MEM (11900024, Gibco) with 20% FCS at 37°C in 5% CO_2_ for 24hrs to allow protein knock-down.

### Phosphorylation site prediction

Potential AurB and Cdk1 phosphorylation sites for Kif4 (accession BAA02167.1) were predicted using GPS 3.0 (freeware, The Cuckoo Workgroup [32–34].

### Statistics

GraphPad Prism 6.0 software (GraphPad Software, Inc) was used for statistical analysis. For numerical data D’Agnostino-Pearson omnibus normality test was performed. For normally distributed data Student’s *t*-test was performed. For data that was not normally disturbed Mann-Whitney test or Kruskal-Wallis Test with Dunn’s post-hoc statistical test was performed. For categorical data Fisher’s Exact Test was used. A p value <0.05 was considered statistically significant.

## Results

### Kif4 has dynamic localization throughout meiosis

**Fig 1:**
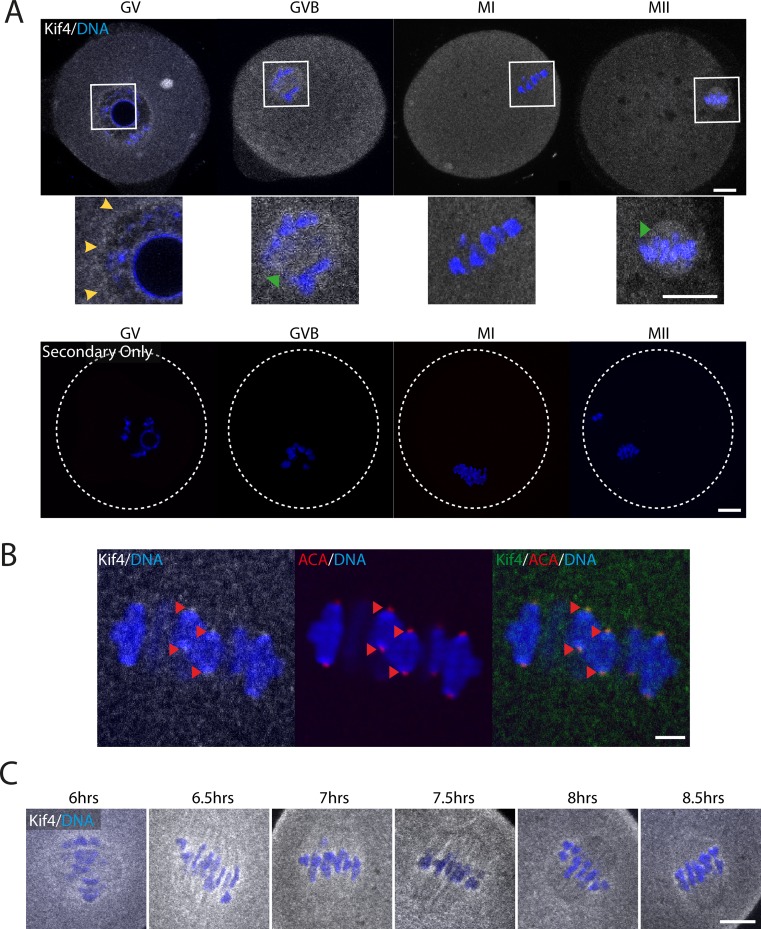
Kif4 has dynamic localization throughout oocyte meiosis. (A) Fluorescent immunolocalization of Kif4 (grey) at GV, GVB, MI and MII. Inserts highlight the localization of Kif4 to the nuclear membrane (yellow arrows) and microtubules (green arrow). Secondary only controls reveal no nonspecific antibody binding. Scale bar = 10μm. (B) Fluorescent immunolocalization of Kif4 (grey/green) to the kinetochore at MI (red arrow). Kinetochores are counter labelled with the inner kinetochore marker ACA (red). Scale bar = 2μm. (C) Fluorescent immunolocalization of Kif4 throughout MI at 6hrs to 8.5hrs post milrinone wash-out. Scale bar = 10μm. DNA is counterstained with Hoechst (blue).

The localization of Kif4 through four distinct stages of female meiosis was investigated, using an antibody directed against the N-terminal motor domain. At each stage examined Kif4 was found dispersed throughout the cytoplasm and in cytoplasm aggregates (Fig 1A). Additionally, it displayed distinct changes in subcellular localization dependent upon meiotic stage. In GV arrested oocytes, Kif4 was found to be enriched around the nuclear envelope and chromatin (Fig 1A). At GVB, Kif4 was distributed around the chromosomes, presumably associated with the microtubule ball, which forms as the meiotic spindle develops (Fig 1A). By MI, Kif4 had relocated and was now associated with the chromosomes where it co-localized with centromere/kinetochore marker ACA (Fig 1B.) To study this change in localization more closely, we analyzed fixed oocytes every 30 minutes throughout late prometaphase-MI and confirmed that, as Kif4 was lost from the microtubules, it become associated with the kinetochore region of the chromosomes (Fig 1C).

Once the polar body was extruded and oocytes had reached MII arrest, Kif4 was once again associated with the microtubules on the metaphase II spindle (Fig 1A). To confirm Kif4 localization throughout meiosis, immunocytochemistry was performed using a second antibody directed against the C-terminal cargo domain with a similar pattern observed (Supplementary S1A Fig).

### Kif4 interacts with both inner and outer kinetochore proteins

**Fig 2:**
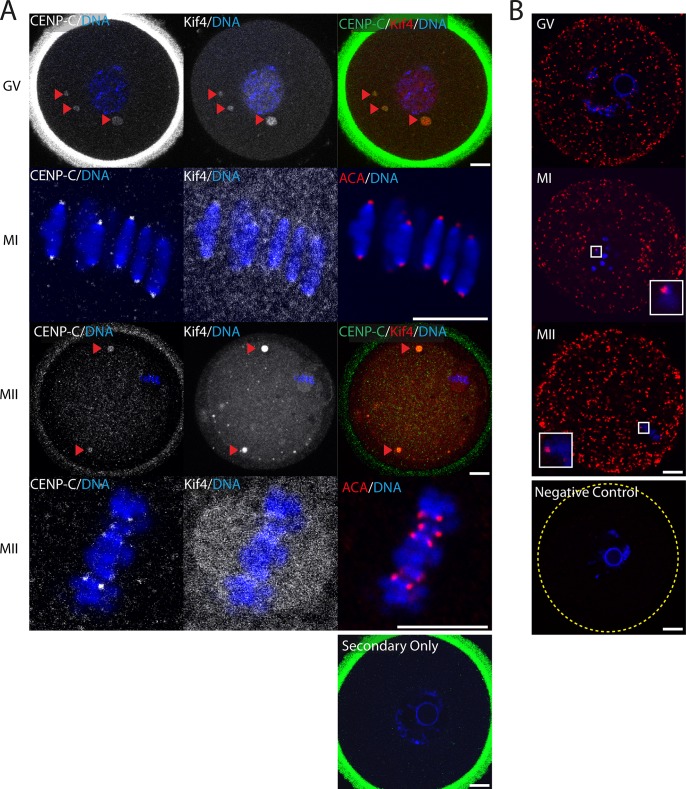
Kif4 co-localizes and interacts with inner kinetochore protein CENP-C throughout meiosis. (A) Fluorescent immunolocalization of Kif4 and CENP-C at GV, MI and MII. MI and MII oocytes are counter labelled with the inner kinetochore marker ACA (red). Red arrows highlight Kif4 and CENP-C aggregates. Secondary antibody only controls reveal nonspecific antibody binding to the zona pellucida only. (B) Positive proximately ligation assay (red) between Kif4 and CENP-C at GV, MI and MII is indicative of protein-protein interaction. Insets highlight interaction on chromosomes (See Supplementary S1B Fig for additional examples). PLA negative control of Kif4 with testis specific Piwil1antibodies reveal no nonspecific signal amplification. DNA is counterstained with Hoechst (blue). Scale bar = 10μm.

Having established that Kif4 was co-located with the centromere/kinetochore at MI, we next sought to investigate whether this kinesin might interact directly with key kinetochore proteins. Immunolocalization revealed Kif4 signal overlap with the inner kinetochore protein, CENP-C at all meiotic stages examined. CENP-C associated with Kif4 aggregates in GV and MII oocytes (Fig 2A) and as expected, CENP-C and Kif4 foci overlapped at the kinetochore, at both MI and MII (Fig 2A). To determine whether these proteins interacted, we performed a proximity ligation assay (PLA) on fixed oocytes using the CENP-C and Kif4 antibodies, and observed positive PLA signal throughout the cytoplasm, at all three stages of meiosis examined (Fig 2B). Furthermore, PLA foci were also associated with the chromosomes, and therefore presumably the kinetochore at MI and MII (Fig 2B and Supplementary S1B Fig).

**Fig 3:**
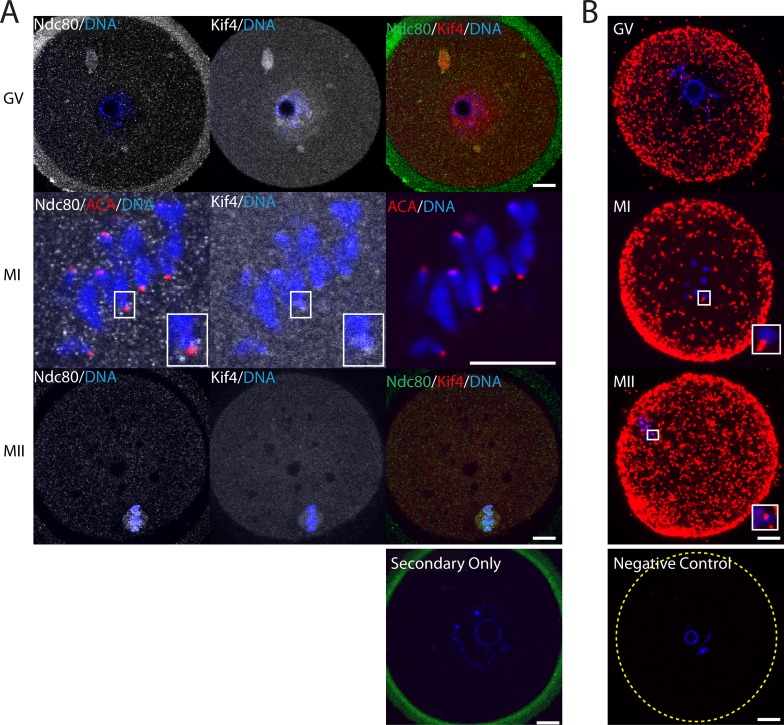
Kif4 co-localizes and interacts with outer kinetochore Ndc80 throughout meiosis. (A) Fluorescent immunolocalization of Kif4 and Ndc80 at GV, MI and MII. MI oocytes are counter labelled with the inner kinetochore marker ACA (red). Secondary only controls reveal nonspecific antibody binding to the zona pellucida only. (B) Positive proximately ligation assay (red) between Kif4 and Ndc80 at GV, MI and MII is indicative of protein-protein interaction. Insets highlight interaction on chromosomes (See Supplementary S1D Fig for additional examples). PLA negative control of Kif4 with testis specific Piwil1 antibodies reveal no nonspecific signal amplification. DNA is counterstained with Hoechst (blue). Scale bar = 10μm.

To gain further insight into the role of Kif4 at the kinetochores, we next investigated its interaction with the outer kinetochore protein Ndc80. As with CENP-C, Ndc80 co-localized with Kif4 aggregates throughout GV oocytes (Fig 3A). At MI Kif4 foci overlapped with ACA fluorescence, however, Ndc80 foci flanked either side, indicative of its role as an outer kinetochore protein (Fig 3A and Supplementary S1C Fig). By MII, both Ndc80 and Kif4 were found on the metaphase spindle. Confirmation of a potential protein-protein interaction was achieved via PLA, with proteins interacting cytoplasmically throughout meiosis (Fig 3B). Additionally, as observed previously, these PLA foci were associated with chromosomes at MI and MII (Fig 3B and Supplementary S1D Fig).

### Kif4 is essential for normal oocyte meiosis

**Fig 4:**
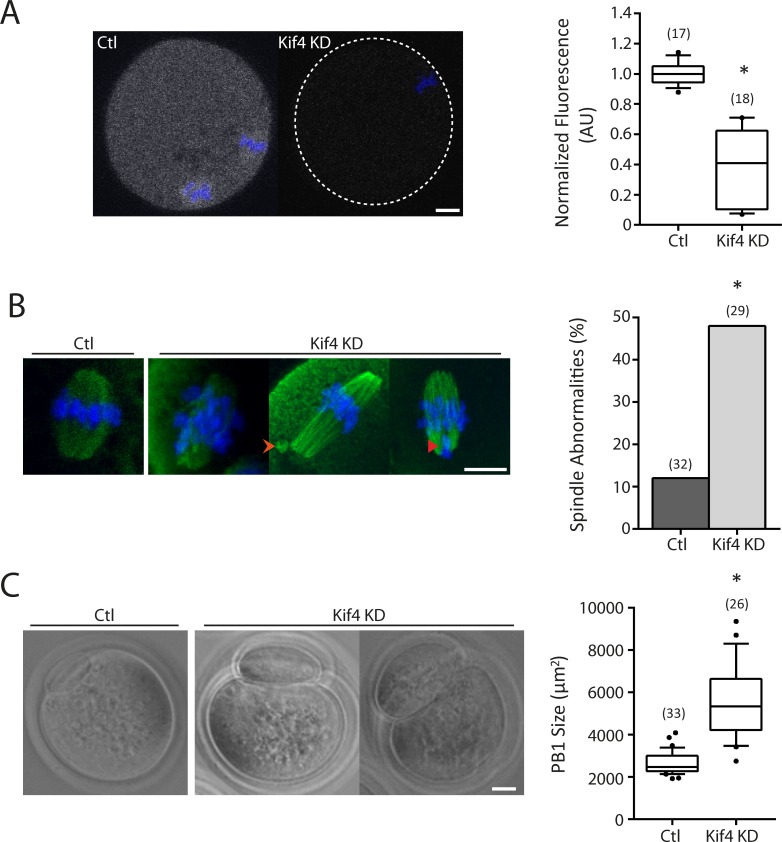
Kif4 is essential for correct spindle formation and cytokinesis. (A) Fluorescent immunolocalization of Kif4 (grey) in control and knock-down (KD) oocytes. Graphical representation of normalized Kif4 fluorescence; p<0.0001, Mann-Whitney test. (B) Fluorescent immunolocalization of α-tubulin at MII in control and Kif4 KD. Kif4 KD oocytes were found to have a higher percentage of abnormal spindles (orange arrow) and chromosome misalignment (red arrow). Graphical representation of abnormal spindle percentages per group; p = 0.0017, Fishers Exact test. (C) Phase contrast images of control and Kif4 KD MII oocytes. Graphical representation of PB1 size between control and Kif4 KD oocytes; p<0.0001, Mann-Whitney test. Box plots show mean (centerline) with box outline 25-75^th^ percentiles and whiskers 10-90^th^ percentiles, bar graphs show mean. n = number of oocytes examined from 3 replicates. DNA is counterstained with Hoechst (blue), scale bar = 10μm.

The spindle/kinetochore localization and potential binding partners of Kif4 suggested it may be important for meiotic progression. To examine its role more closely Kif4 knock-down (KD) was performed via oligo morpholino translational repression. Using immunocytochemistry we confirmed Kif4 protein expression was reduced 2.5 fold 24hrs post morpholino injection when compared to mis-match morpholino injected controls (0.39±0.25 AU KD v 1±0.07 AU control, p<0.0001; Fig 4A). IVM of KD oocytes found no abnormalities in meiosis timing or maturation rates (data not shown). However, investigation of MII oocyte quality found KD oocytes had a significantly higher proportion of abnormal MII spindles compared to controls (48% knock-down v 12% controls, p = 0.0017; Fig 4B). Spindle abnormalities included mild and severe chromosome misalignment, and microtubule aggregates attached to spindles. Additionally, KD oocytes were observed to have abnormal cytokinesis as determined by a significant increase in polar body size (5489±1692μm^2^ KD v 2661±522μm^2^ control, p<0.0001; Fig 4C).

### Kif4 and kinetochore protein interaction is mediated via kinase activity

**Fig 5:**
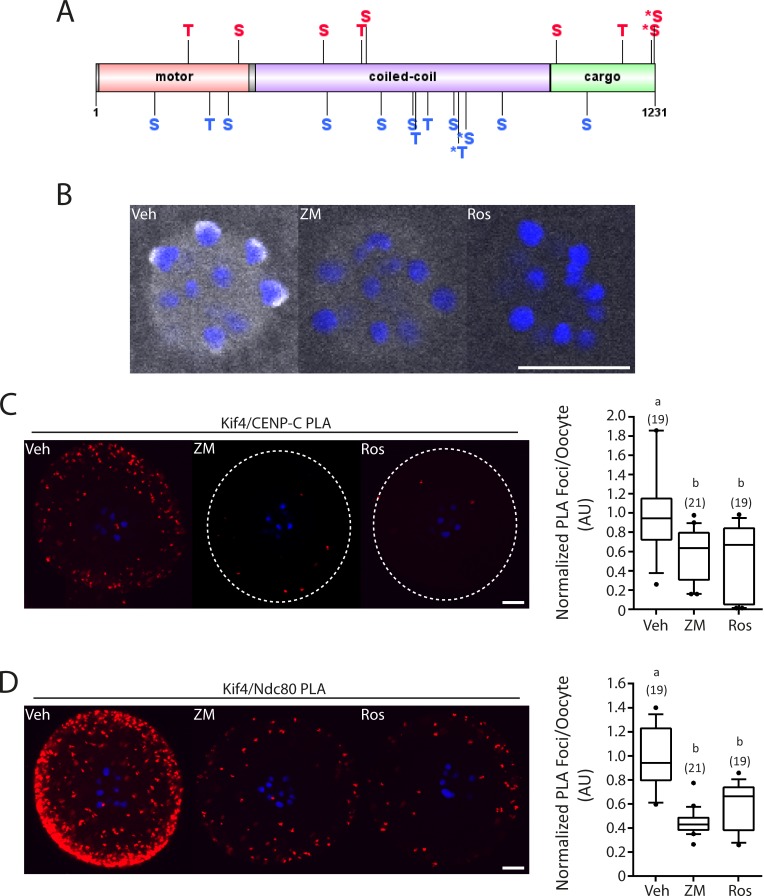
Kinase inhibition reduces Kif4 kinetochore protein interaction. (A) Schematic representation of mouse Kif4 including motor (pink), coiled-coil (purple) and cargo (green) domains. Serine (S) and threonine (T) predicated phosphorylation sites for Cdk1 (red) and AurB (blue) are labelled. * indicates known Kif4 phosphorylation sites. (B) Fluorescent immunolocalization of Kif4 (grey) at MI following 3.5hrs of treatment with vehicle (Veh) DMSO, aurora kinase inhibitor ZM 447439 (ZM) or CDK inhibitor Roscovitine (Ros). (C) PLA (red) of Kif4 and CENP-C at MI following 3.5hrs of treatment with the Veh, ZM or Ros. Graphical representation of normalized PLA foci per oocyte; p = 0.0013, Kruskal-Wallis with Dunn’s Post-hoc. (D) PLA (red) of Kif4 and Ndc80 at MI following 3.5hrs of treatment with the Veh, ZM or Ros. Graphical representation of normalized PLA foci per oocyte; p<0.00031, Kruskal-Wallis with Dunn’s Post-hoc. Box plots show mean (centerline) with box outline 25-75^th^ percentiles and whiskers 10-90^th^ percentiles; n = number of oocytes examined from 3 replicates. DNA is counterstained with Hoechst (blue). Scale bar = 10μm.

Since meiosis is driven by the activity of several important kinases, we next sought to determine if the function of Kif4 was regulated in this manner. Previous research has found that Cdk1 and AurB mediate the interaction of Kif4 with condensin I [19]. Furthermore, AurB has been found to directly interact with Kif4 at mitotic midzones [14]. Group based prediction of AurB and Cdk1 phosphorylation sites on Kif4 found 9 potential Cdk1 sites and 13 potential AurB sites located throughout the three major domains; motor, coiled-coil and cargo (Fig 5A). Of note is that 4 of these sites, serine 816, 1224, 1230 and threonine 800, are known Kif4 phosphorylation residues [35]. Additionally threonine 800 is a well categorized AurB phosphorylation site [14,21].

To examine whether Cdk1 and AurB kinase activity did in fact regulate Kif4, oocytes were allowed to mature in the presence of either the pan Aurora Kinase inhibitor ZM 447439 (ZM) or the pan Cdk inhibitor Roscovitine (Ros) for 4hrs from prometaphase to MI. Kif4 cytosolic immunolocalization was unchanged with Cdk and Aurora Kinase inhibition. However, immunolocalization of Kif4 to the kinetochore was lost after treatment with both ZM and Ros suggesting an inability of Kif4 to relocalize to the kinetochore with Cdk and/or Aurora Kinase inhibition (Fig 5B). Furthermore, PLA analysis found that both treatments significantly reduced Kif4/CENP-C and Kif4/Ndc80 interaction compared to the vehicle control (CENP-C: 0.58±0.27 PLA foci ZM v 0.54±0.37 PLA foci Ros v 1±0.50 PLA foci Veh, p = 0.0013; Fig 5C and Ndc80: 0.44±0.12 PLA foci ZM v 0.60±0.19 PLA foci Ros v 1±0.25 PLA foci Veh, p<0.0001; Fig 5D).

To confirm that these results were the consequence of specific Aurora Kinase or Cdk regulation oocytes were allowed to mature in the presence of the Plk1 inhibitor BI 2536. Unlike Ros or ZM treatment, BI 2536 had no effect on Kif4 kinetochore localization consistent with the lack of Plk1 and Kif4 co-localization off the kinetochores (Supplementary S2 Fig).

### Kif4 expression increases with maternal ageing

**Fig 6:**
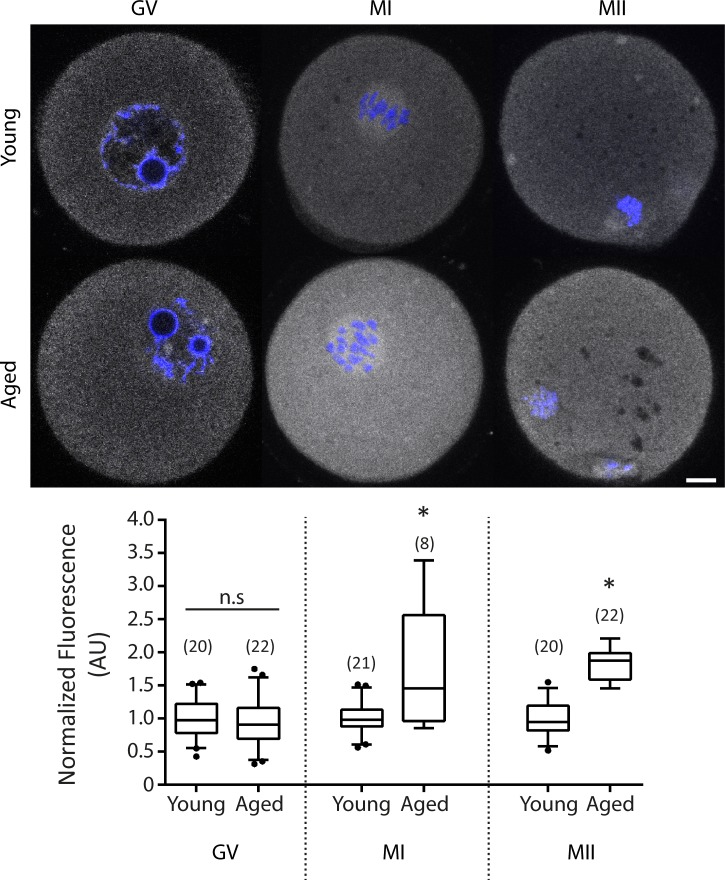
Kif4 protein expression increases with maternal ageing. Fluorescent immunolocalization of Kif4 (grey) in GV, MI and MII oocytes from young and maternally aged animals. Graphical representation of normalized Kif4 fluorescence at GV (p = 0.6891, Student’s *t*-test), MI (p = 0.0025, Student’s *t*-test) and MII (p<0.0001, Student’s *t*-test). Box plots show mean (centerline) with box outline 25-75^th^ percentiles and whiskers 10-90^th^ percentiles; n = number of oocytes examined from 4 animals. DNA is counterstained with Hoechst (blue). Scale bar = 10μm.

Finally, we sought to determine if Kif4 protein was altered with maternal age in oocytes which might be associated with their reduced quality. Immunocytochemistry revealed that Kif4 expression was unchanged in young vs aged GV stage oocytes (0.95±0.39 AU Aged v 1±0.28 AU Young, p = 0.6891; Fig 6). However, by MI there was a significant increase in protein expression (1.73±0.93 AU Aged v 1±0.26 AU Young, p = 0.0025; Fig 6) which continued into MII (1.81±0.28 AU Aged v 1±0.28 AU Young, p<0.0001; Fig 6). Quantitative gene expression (qRT-PCR) showed no change in transcript level at GV (p = 0.0741; Supplementary S3A Fig) or MII (p = 06964; Supplementary S3B Fig) indicating the increase of Kif4 may have been a result of elevated translation or reduced protein turnover with age.

## Discussion

In the current study we have demonstrated an essential role for Kif4 in mammalian oocyte meiosis and highlighted its potential interaction with key kinetochore proteins and regulation by meiotically important kinases.

The localization of Kif4 in oocytes is dynamic: we observed that as meiosis I progressed Kif4 left the metaphase I spindle and became enriched on the chromosomes and kinetochores. This enrichment at the kinetochore towards the end of metaphase I occurs at time when kinetochore-microtubule (KT-MT) attachments are stabilized and so could indicate a role for Kif4 in this stabilization. In line with this, is the known role of Kif4 in microtubule stabilization in mitotic cells and *Xenopus* egg extracts. Ablation of Kif4 from cells lines or XKlp1 from *Xenopus* egg extracts significantly altered microtubule dynamics including microtubule overgrowth, decreased KT-MT flux and altered kinetochore oscillations [7,27,36,37]. These altered microtubule dynamics ultimately lead to abnormal spindle formation and misaligned chromosomes, cumulating in aneuploidy with misaligned chromosomes as observed in our KD oocytes [9,36,37]. Evidence in mitotic extracts suggests that the abnormal microtubule dynamics is a likely cause of XKlp1 limiting microtubule growth via allosteric inhibition of microtubule dynamic instability [38]. Alterations in microtubule stability, growth and kinetochore oscillations could account for the metaphase spindle abnormalities observed in KD oocytes. Increased microtubule length, and reduced kinetochore oscillations would be expected to cause the chromosome misalignment we observed. Further to this, microtubule overgrowth was detected in Kif4 ablated oocytes, with the addition of microtubule spheres attached to spindle poles.

Cytokinesis abnormalities were also a common feature in Kif4 KD oocytes. Ablation of Kif4, XKlp1 or Klp3A mutation results in cytokinesis failure in HeLa cells, mitotic *Xenopus* egg extracts or *Drosophila* spermatocytes respectively [15,24,37]. Conversely, we have shown that depletion of Kif4 in oocytes results in abnormal cytokinesis as demonstrated via enlarged polar bodies. This indicates that Kif4 is not essential for cytokinesis but is necessary for asymmetric cytokinesis in oocytes.

Interestingly, enlarged polar bodies have previously also been observed in oocytes depleted of Ndc80 [39]. Of note is that Ndc80 is crucial for correct spindle formation and chromosome alignment at metaphase I and II in mouse and porcine oocytes [39–41]. The similar phenotype observed between Ndc80 and Kif4 ablated oocytes in conjunction with their continued co-localization and potential interaction throughout meiosis highlights a probable role for Kif4 in correct Ndc80 function throughout the cell cycle. Interestingly, Kif4 was also found to interact with CENP-C throughout meiosis. KD of CENP-C in DT40 chicken cells results in abnormal chromosome alignment similar to that seen in our Kif4 KD oocytes [42]. It is therefore likely that Kif4 depletion from oocytes results in alterations to both CENP-C and Ndc80 localization, which may be responsible for the observed abnormal metaphase II oocyte phenotype.

It is also tempting to speculate that CENP-C and Ndc80 are potential cargo proteins of Kif4, with Kif4 involved in the correct shuttling of these proteins throughout the oocyte. In support of this theory, KD of Kif4 in DT40 cells significantly altered the expression of a large number of kinetochore proteins, including CENP-C, Ndc80 and AurB specifically on chromosomes [11]. We found that interaction of kinetochore proteins CENP-C or Ndc80 with Kif4 was not restricted to chromosomes, but was also found throughout the cytoplasm of meiotically cycling oocytes. As kinesins have traditionally been associated with roles in cargo shuttling, it is possible that Kif4 is at least partially responsible for shuttling CENP-C/Ndc80 to and from the chromosomes. Ablation of Kif4, therefore, could result in incorrect loading of CENP-C/Ndc80 to the kinetochore which might account for the chromosome misalignment observed.

Intriguingly, the movement of Kif4 to the kinetochores or its interaction with CENP-C and Ndc80 appears to be cell cycle dependent and under the control of Aurora Kinase and Cdk activity. CENP-C and Ndc80 expansion has been found to be AurB dependent and essential for correct KT-MT attachments [43]. Further to this, AurB is responsible for phosphorylation of human Kif4 T799 (mouse T800), with an additional12 predicted AurB phosphorylation sites identified [21]. Oocytes express Aurora Kinase B and C, with both inhibited with ZM. AurB/C is essential in meiosis I for correct KT-MT attachment and spindle formation, with AurB/C destabilizing KT-MT attachments to allow error correction [44–46]. The phosphatase PP2A is a known antagonist of AurB and has also been found to interact with Kif4, reversing T799 phosphorylation [21]. Furthermore, Kif4 has been found to regulate PP2A localization throughout mitosis [21]. In contrast, Cdk1 appears to regulate KT-MT attachment stabilization [47]. Cdk1 activity increases throughout meiosis I with Cdk1 inhibition during prometaphase/metaphase I leading to a reduction in stable KT-MT attachments [47]. Conversely, over activation of Cdk1 leads to accelerated stabilization of KT-MT during meiosis I [47]. It is therefore possible that, AurB and Cdk1 recruits Kif4 to the kinetochore during the later stages of prometaphase when microtubule stabilization is occurring. This accumulation, could in turn recruit PP2A. Stabilization of correct KT-MT attachments via Kif4 directly, and PP2A dephosphorylation of AurB/C substrates could then occur. In support of this, KD of Kif4 in oocytes led to a significant increase in spindle abnormalities including misaligned chromosomes, likely a result of incorrect KT-MT attachments. In addition, the localization of Kif4 to the kinetochore and its interaction with kinetochore proteins appears to be under kinase control.

It is important to note, however, that inhibitors used throughout this study are pan Cdk and Aurora Kinase inhibitors. Research into the role of other cyclin dependent kinases in oocyte meiosis is limited, however it appears probable that Cdk1 is the key Cdk in oocyte meiosis [48]. As Cdk1 has been found in numerous studies to interact with Kif4, it is also the most likely candidate for Kif4 regulation in oocytes [19,20]. Additionally, to date there is evidence for AurB but not AurC, directed regulation of Kif4. ZM inhibits both AurB and AurC and unlike somatic cells AurC regulation has been found to have essential roles in oocyte meiosis, with multiple proteins being controlled via both AurB and C kinases [49]. Therefore it cannot be ruled out that AurC may be involved in Kif4 functioning throughout meiosis, although this requires further investigation.

Finally, maternal ageing is a well-established cause of reduced oocyte quality, which ultimately leads to subfertility [2]. Considering the overexpression of Kif4 at the protein level in reproductively aged mice at the MI/MII stage, this indicates a potential role for Kif4 in age related oocyte quality decline. Interestingly, aged GV oocytes had normal Kif4 levels, suggesting that Kif4 upregulation occurs as a result of its increased translation or reduced turnover post meiotic resumption. This increased expression of Kif4, a known microtubule stabilizing protein, could result in increased microtubule stabilization throughout meiosis in aged oocytes. In support of this, Kif4 overexpression in migrating fibroblasts has been shown to increase the number of microtubules resistant to the microtubule destabilizing agent nocodazole—consistent with a role for Kif4 in microtubule stabilization [50]. Furthermore, maternally aged oocytes are less sensitive to nocodazole treatment, with aged oocytes having increased ability to complete meiosis I in the presence of nocodazole compared to young oocytes [51]. Additionally, maternally aged oocytes have a higher frequency of KT-MT attachment errors, ultimately leading to aneuploidy [4]. It is therefore possible that over-expression of Kif4 with age partially desensitizes oocytes to spindle abnormalities and incorrect KT-MT attachment. However, further investigation is needed to determine the potential cause and consequence of Kif4 upregulation with maternal age.

In conclusion we have found that Kif4 is expressed throughout oocyte meiosis and has essential roles in cytokinesis and spindle formation. Furthermore, Kif4 localization and interaction with kinetochore proteins appears to be regulated via AurB and Cdk1. In addition, its upregulation with age makes Kif4 a promising lead protein in our understanding of age-related oocyte quality decline.

## Supporting Information

S1 Fig (A) Immunolocalization of Kif4 at GV, GVB, MI and MII with C-terminal direct antibody. (B) Kif4 and CENP-C PLA foci in MI oocytes were found throughout the cytoplasm and associated with chromosomes (yellow arrow). (C) Fluorescent immunolocalization of Kif4 and Ndc80 at MI. Oocytes are counter labelled with the inner kinetochore marker ACA (red). (D) Kif4 and Ndc80 PLA foci in MI oocytes were found throughout the cytoplasm and associated with chromosomes (yellow arrow). DNA is counterstained with Hoechst (blue). Scale bar = 10μm (A, B & D) or 5μm (C).
(TIF)

S2 Fig (A) Immunolocalization of Kif4 and Plk1 at MI at the kinetochores and spindle poles (B) Fluorescent immunolocalization of Kif4 (grey) at MI following 3.5hrs of treatment with the vehicle (Veh) DMSO or Plk1 inhibitor BI 2536. Scale bar = 10μm.
(TIF)

S3 Fig (A) Relative expression (ΔCt) of Kif4 mRNA between young and aged females in GV oocytes; p = 0.0741, Student’s *t*-test. (B) Relative expression (ΔCt) of Kif4 mRNA between young and aged females in MII oocytes; p = 06964, Student’s *t*-test. Bar graphs show mean with SD marked, n = number of animals.
(TIF)

S1 Table Antibodies used for immunocytochemistry and proximity ligation. (DOCX)

S2 Table Morpholino target sequence. (DOCX)
