# Supplementary figures and images for: Kif4 Is Essential for Mouse Oocyte Meiosis
Source: PLoS One. 2017 Jan 26;12(1):e0170650. doi: 10.1371/journal.pone.0170650 (PMC5268449; doi:10.1371/journal.pone.0170650)

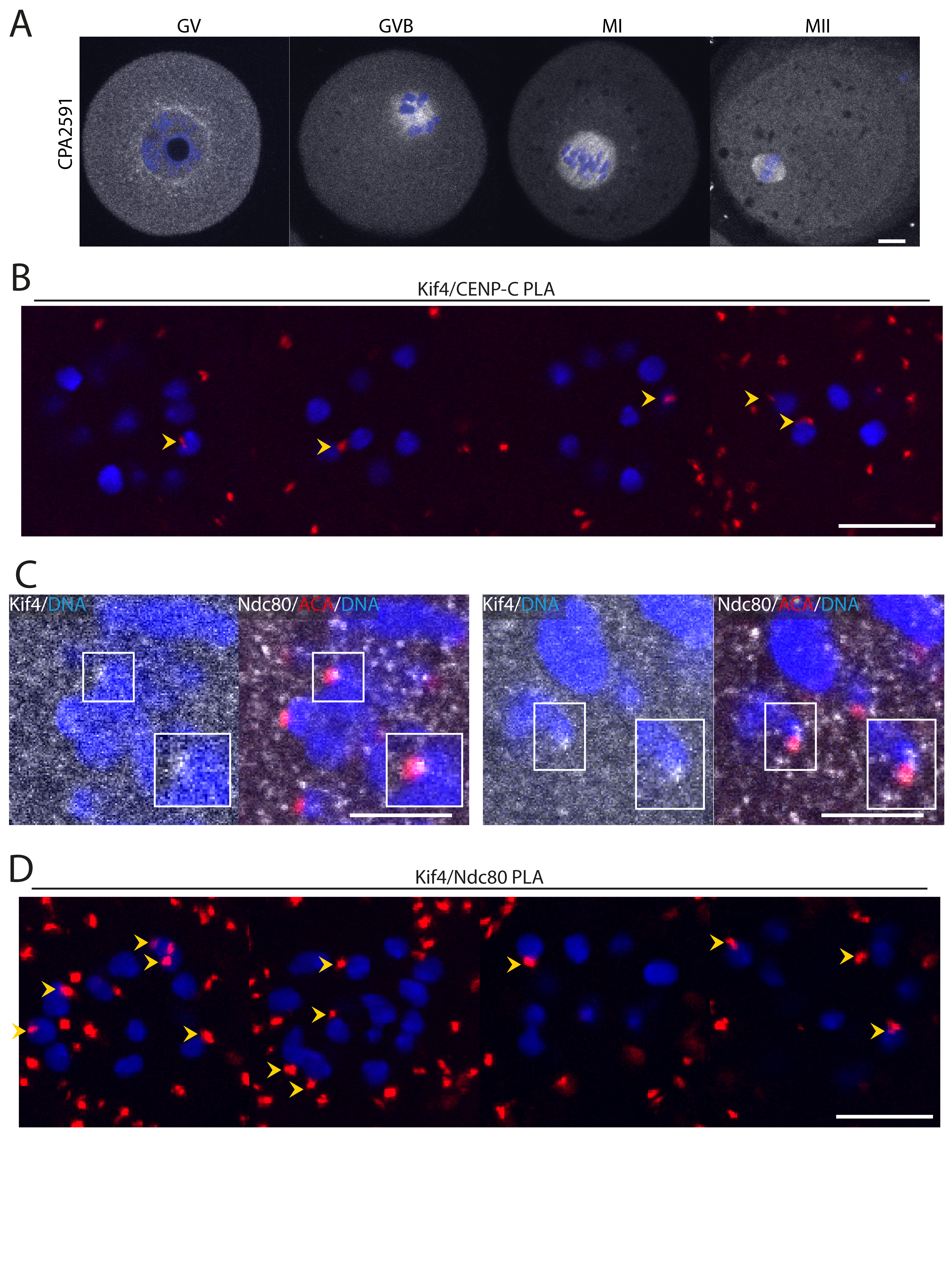

Supplement: S1 Fig — (A) Immunolocalization of Kif4 at GV, GVB, MI and MII with C-terminal direct antibody. (B) Kif4 and CENP-C PLA foci in MI oocytes were found throughout the cytoplasm and associated with chromosomes (yellow arrow). (C) Fluorescent immunolocalization of Kif4 and Ndc80 at MI. Oocytes are counter labelled with the inner kinetochore marker ACA (red). (D) Kif4 and Ndc80 PLA foci in MI oocytes were found throughout the cytoplasm and associated with chromosomes (yellow arrow). DNA is counterstained with Hoechst (blue). Scale bar = 10μm (A, B & D) or 5μm (C). (TIF) [file pone.0170650.s001.tif]

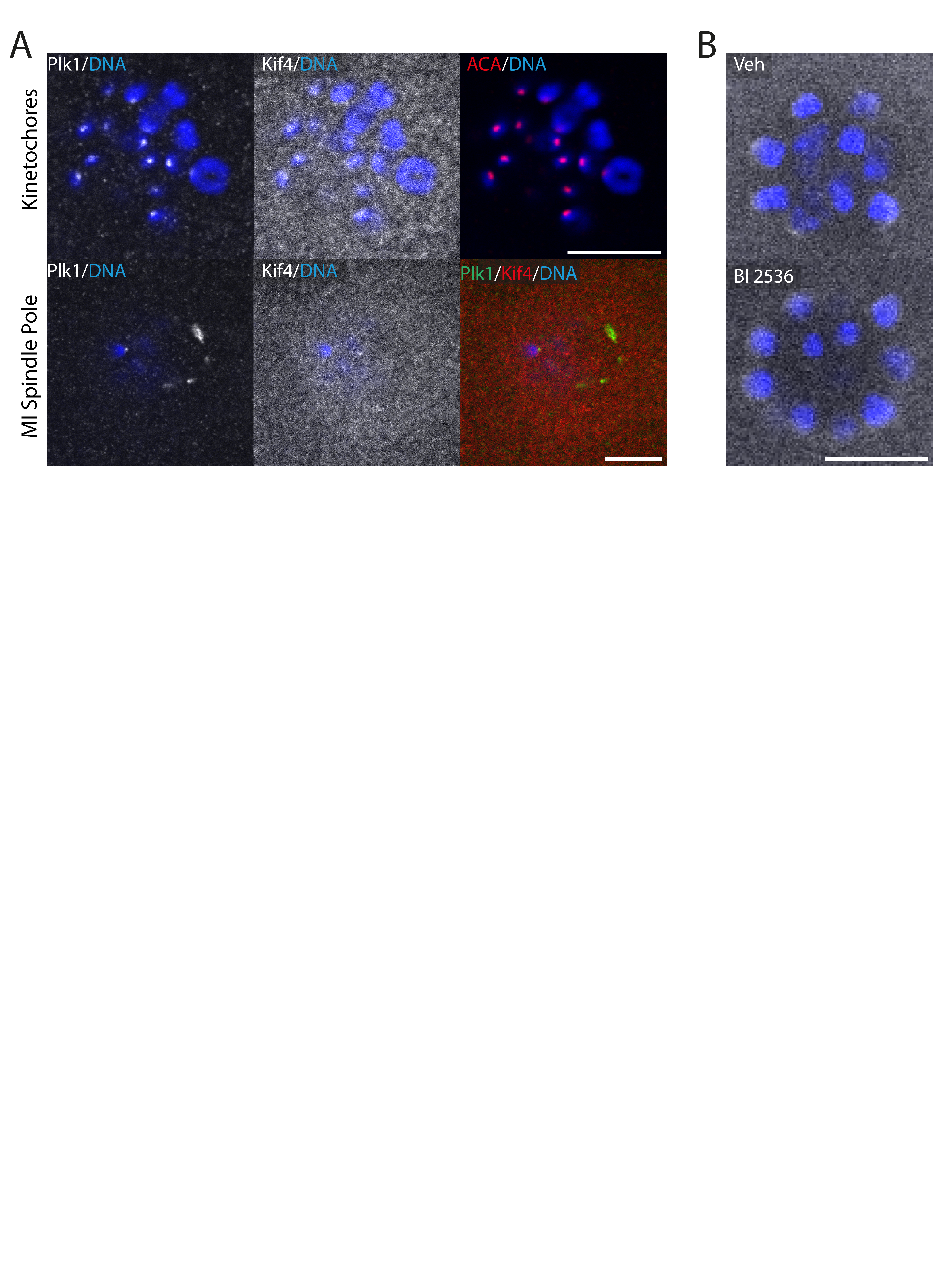

Supplement: S2 Fig — (A) Immunolocalization of Kif4 and Plk1 at MI at the kinetochores and spindle poles (B) Fluorescent immunolocalization of Kif4 (grey) at MI following 3.5hrs of treatment with the vehicle (Veh) DMSO or Plk1 inhibitor BI 2536. Scale bar = 10μm. (TIF) [file pone.0170650.s002.tif]

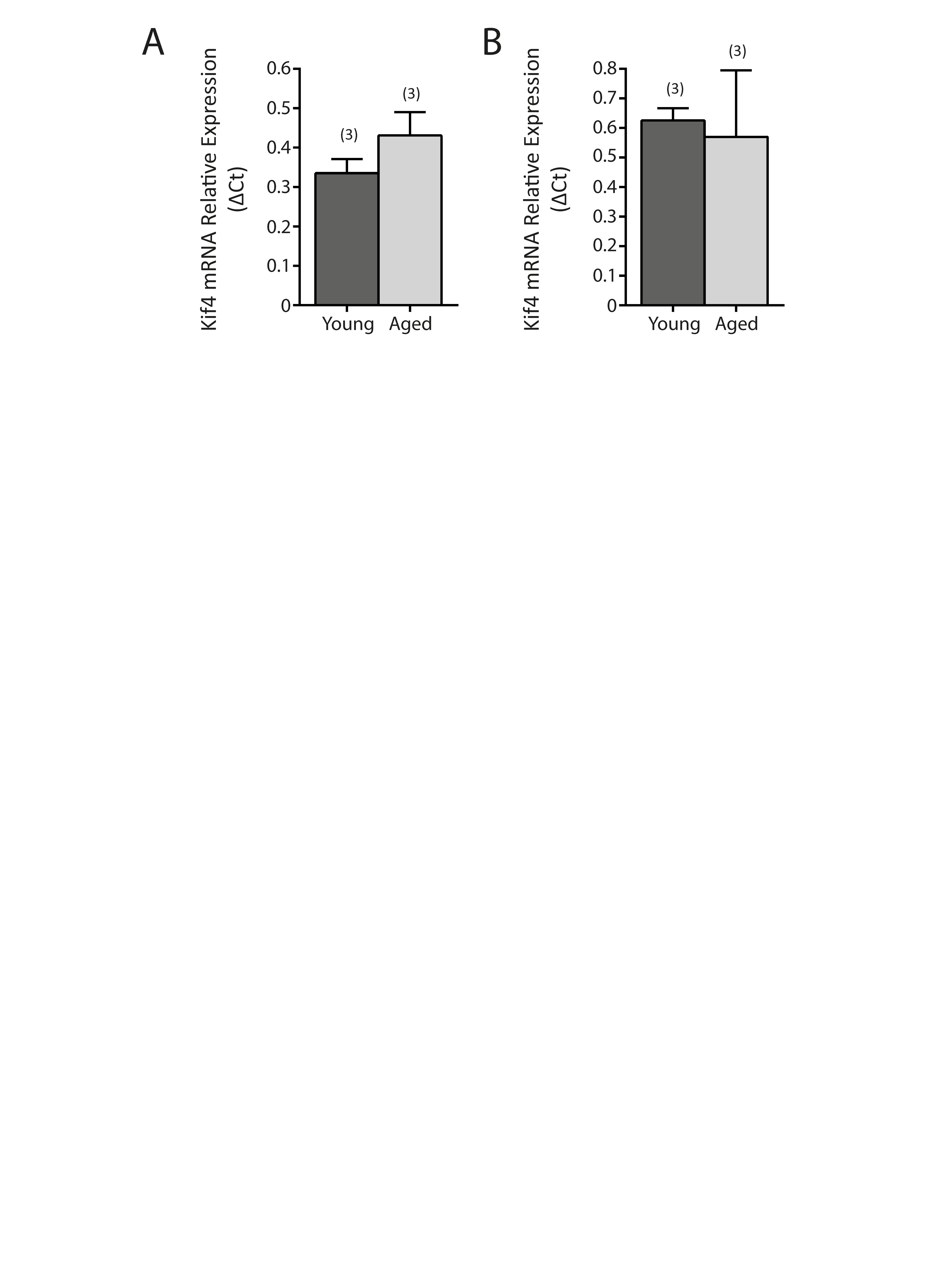

Supplement: S3 Fig — (A) Relative expression (ΔCt) of Kif4 mRNA between young and aged females in GV oocytes; p = 0.0741, Student’s t-test. (B) Relative expression (ΔCt) of Kif4 mRNA between young and aged females in MII oocytes; p = 06964, Student’s t-test. Bar graphs show mean with SD marked, n = number of animals. (TIF) [file pone.0170650.s003.tif]
