# Supplementary material for: Kif4 Is Essential for Mouse Oocyte Meiosis
Source: PLoS One. 2017 Jan 26;12(1):e0170650. doi: 10.1371/journal.pone.0170650 (PMC5268449; doi:10.1371/journal.pone.0170650)
Supplement: S1 Table — (DOCX) [file pone.0170650.s004.docx]

**S1 Table: Antibodies used for immunocytochemistry and proximity ligation**

| **Antibody** | **Species Raised In** | **Dilution** | **Source and Cat. #** |
| --- | --- | --- | --- |
| Kif4a (N-terminal) | Rabbit (Polyclonal) | 1:100 | Abcam (ab72337) |
| Kif4a (C-terminal) | Rabbit (Polyclonal) | 1:400 | Cohesion Biosciences (CPA2591) |
| Ndc80 | Mouse (Monoclonal) | 1:250 | Abcam (ab3613) |
| CENP-C | Mouse (Monoclonal) | 1:250 | Abcam (ab50974) |
| Anti-centromere Antibody | Human (Polyclonal) | 1:400 | Fitzgerald (90C-CS1058) |
| Anti-α tubulin | Mouse (Monoclonal) | 1:400 | Invitrogen (A-11126) |
| Anti-mouse 488 | Goat (Polyclonal) | 1:1000 | Invitrogen (A-11001) |
| Anti-human 555 | Goat (Polyclonal) | 1:1000 | Invitrogen (A-21433) |
| Anti-rabbit 633 | Goat (Polyclonal) | 1:1000 | Invitrogen (A-21070) |
