# Supplementary material for: Kif4 Is Essential for Mouse Oocyte Meiosis
Source: PLoS One. 2017 Jan 26;12(1):e0170650. doi: 10.1371/journal.pone.0170650 (PMC5268449; doi:10.1371/journal.pone.0170650)
Supplement: S2 Table — (DOCX) [file pone.0170650.s005.docx]

**S2 Table:**  **Morpholino target sequence**

| **Morpholino** | **Sequence** |
| --- | --- |
| Knock-down | TCCCCTTCACCTCTTCTTTCATGGT |
| Mismatch | TCaCCTTaACCTaTTCTTTaATcGT |
